# Atypical imaging characteristics in patients with epithelial tumors of lacrimal gland and impact on initial surgical planning

**DOI:** 10.3389/fonc.2026.1852743

**Published:** 2026-07-23

**Authors:** Bita Esmaeli, Tracy Lu, Leena Ketonen, James Matthew Debnam

**Affiliations:** 1 Orbital Oncology and Ophthalmic Plastic Surgery, Department of Plastic Surgery, The University of Texas MD Anderson Cancer Center, Houston, TX, United States; 2 Oncologic Ophthalmic Plastic & Orbital Surgery, Mann Eye Institute, Houston, TX, United States; 3 Department of Clinical Sciences, University of Houston Fertitta Family College of Medicine, Houston, TX, United States; 4 Department of Ophthalmology, Houston Methodist Hospital/Weill Cornell Medical College, Houston, TX, United States; 5 Department of Neuroradiology, The University of Texas MD Anderson Cancer Center, Houston, TX, United States

**Keywords:** atypical imaging findings, gross total resection, lacrimal gland carcinoma, magnetic resonance imaging, pleomorphic adenoma

## Abstract

**Precis:**

Approximately one-quarter of lacrimal gland masses present with imaging findings not consistent with standard teaching for benign vs. malignant; it is prudent to do gross total resection in such cases if eye-sparing surgery is possible.

**Background:**

Epithelial lacrimal gland tumors are rare. The initial surgical decision to do an incisional biopsy vs. gross total resection depends largely on the radiologic characteristics of the lacrimal gland mass. Occasionally, the reported radiologic features attributed to benign vs. malignant lacrimal gland tumors do not hold. In this report, we will focus on these atypical presentations to help the orbital surgeon with better surgical planning.

**Methods:**

Epithelial lacrimal gland tumors diagnosed in 101 consecutive patients (59 adenoid cystic carcinomas, 24 other carcinomas, and 18 pleomorphic adenomas) and treated by the primary author were included. The clinical and radiologic records were retrospectively reviewed. The clinical parameters analyzed included age, gender, histologic type, and surgical treatment. The radiologic data analyzed included radiologic characteristics of the lacrimal gland mass on the initial magnetic resonance imaging (MRI) and/or computed tomography (CT) at presentation. Cases that did not follow the “standard” imaging characteristics of carcinoma or pleomorphic adenoma were further analyzed.

**Results:**

Overall, two possible atypical radiologic patterns were identified: (1) 11 of 59 (19%) cases of adenoid cystic carcinoma and 5 of 24 (21%) cases of other carcinomas were identified where the lesion was round and well-circumscribed, lacked a posterior extension or other aggressive features, and could not be distinguished from pleomorphic adenoma but after surgical resection were found to be adenoid cystic carcinoma or other types of lacrimal gland carcinoma; (2) 5 of 18 (28%) cases of pleomorphic adenoma presented with large multilobular masses and could not be distinguished from a carcinoma but in fact were found to be of benign nature on histologic evaluation.

**Conclusions:**

Our findings suggest some overlap in radiologic features between pleomorphic adenomas and lacrimal gland carcinomas. Given the risk of multifocal benign or malignant recurrence of pleomorphic adenomas if excised incompletely, it seems prudent to err on the side of gross total resection for most well-circumscribed lesions of the lacrimal gland that lack aggressive radiologic features. Some of these well-circumscribed lesions will end up being adenoid cystic carcinoma or other types of lacrimal gland carcinomas upon pathologic evaluation of the surgical specimen and may require additional treatments.

## Introduction

Epithelial lacrimal gland tumors are rare (1, 2). The initial surgical decision to do an incisional biopsy vs. gross total resection (“lumpectomy”) depends largely on the radiologic characteristics of the lacrimal gland mass and the presenting clinical symptoms and signs (1–3).

Pleomorphic adenoma (PA) usually presents as a slow-growing mass causing painless proptosis and downward displacement of the eye, and in some cases, slow gradual visual loss due to pressure effect on the eye. On magnetic resonance imaging (MRI), PAs are usually well-circumscribed, round to oval, isointense lesions in the lacrimal gland fossa with smooth margins, with typical remodeling and expansion of the bony walls without destruction (4). Recurrent PA tends to be multinodular and multifocal (5, 6). In cases of malignant transformation to carcinoma, the lesion presents with a heterogeneous appearance, infiltration of adjacent soft tissue, and possible bony destruction (5). In general, when a PA is the preoperative clinical diagnosis, a complete surgical excision of the lacrimal gland mass with an intact capsule is the appropriate surgery. Violation of the capsule can lead to benign multifocal recurrences that lead to multiple surgeries with associated ocular morbidity and concern for malignant transformation to carcinoma ex PA.

In contrast, lacrimal gland carcinomas usually present as a more rapidly growing mass compared with PA, are more frequently associated with pain, extend posteriorly and laterally along the lateral wall of the orbit toward the superior orbital fissure (on MRI), and are more likely to infiltrate into the intraconal space (3, 7). In locally advanced cases, bony erosion, dural and CNS extension, and involvement of the temporal fossa are possible (8, 9). The appropriate initial surgical procedure for a suspected lacrimal gland carcinoma may be an incisional biopsy. Once the diagnosis of carcinoma is confirmed, additional treatments can be planned such as neoadjuvant chemotherapy or targeted therapy, additional eye-sparing surgery vs. orbital exenteration, and the patient can be counseled regarding adjuvant treatments (10).

Occasionally, the reported radiologic features attributed to benign vs. malignant epithelial tumors of the lacrimal gland do not follow the expected patterns. This can create dilemmas in decision-making by the orbital surgeon, specifically whether to do an incisional biopsy vs. gross total resection of the lacrimal gland mass as the initial surgical intervention. In this report, we evaluate the frequency of atypical radiologic presentations in a relatively large cohort of patients with lacrimal gland epithelial tumors and explore the implications of this finding in terms of the choice of initial surgical intervention by the orbital surgeon.

## Patients and methods

All consecutive patients who had a diagnosis of primary epithelial tumor of lacrimal gland (PAs and lacrimal gland carcinomas of any histologic type) and were treated by the primary author (B.E.) during the period from November 1998 through August 2024 were included. The medical records, including radiologic records of all eligible patients, were retrospectively reviewed. The eligibility criterion for inclusion in the study was a diagnosis of primary epithelial lacrimal gland tumor (benign or malignant) as confirmed by surgical pathology. Only patients for whom reliable radiologic images before surgery were available for analysis were included. The clinical parameters analyzed included age, gender, date of initial diagnosis, and histopathologic diagnosis. The radiologic data analyzed included radiologic characteristics of the lacrimal gland mass on the initial MRI and/or computed tomography (CT). The specific radiologic parameters analyzed included size and extent of the mass, whether or not the lacrimal gland mass was well-circumscribed or infiltrative, did it have a smooth round to mildly lobulated contour (typical of PA) or was it infiltrative (typical of carcinoma), and whether or not there were bony changes that suggested aggressive behavior such as bony erosion through the lacrimal gland fossa and extension beyond the bony walls of the orbit. Cases that did not follow the expected imaging characteristics of carcinoma or PA, in other words cases that were thought to be PA but turned out to be carcinoma based on surgical pathology or vice versa, were further analyzed. For lacrimal gland carcinomas, atypical imaging findings on preoperative imaging were defined as a round and well-circumscribed lesion that lacked a posterior extension along the superior and lateral orbit and lacked bony erosion. Atypical findings for PA included marked heterogeneity, or the presence of a large multilobular mass rather than the expected well-rounded mildly lobulated smooth mass that is typical for PA.

All images were reviewed by two independent neuroradiologists (J.M.D. and L.K.). The radiologists were blinded to the histologic diagnosis. If there was lack of interobserver agreement, a third neuroradiologist was invited to review the imaging studies.

This research was done in accordance with the Health Insurance Portability and Accountability Act. Institutional Review Board approval was obtained from The University of Texas MD Anderson Cancer Center (protocol number: PA 2024-1244). This research adhered to the tenets of the Declaration of Helsinki.

### Statistical methods

Descriptive statistics were used to describe the patients’ characteristics and clinical outcomes. Continuous variables were summarized using mean, standard deviation, median, and range, while categorical variables were described by frequencies and percentages.

## Results

A total of 101 patients (60 men and 41 women) met the eligibility criteria. The age ranged from 13 to 103 years (median age, 56 years). There were 59 adenoid cystic carcinomas, 24 other carcinomas, and 18 PAs.

Overall, two possible atypical radiologic patterns were identified: (1) The first atypical pattern was found in 11 of 59 (19%) cases of adenoid cystic carcinoma and 5 of 24 (21%) cases of other carcinomas. In these cases, the preoperative radiologic appearance of the lacrimal gland carcinoma was round and well-circumscribed, and the lesion lacked a posterior extension along the lateral and posterior orbit or other aggressive features such as bony erosion or extension beyond the bony confines of the orbit and could not be reliably distinguished from PA. The diagnosis of carcinoma was confirmed histologically after gross total surgical resection despite the preoperative imaging findings suggesting a PA. (2) The second atypical pattern was found in 5 of 18 (28%) cases of PA, which presented as large multilobular masses that could not be reliably distinguished from a lacrimal gland carcinoma and upon histopathologic evaluation of the surgical specimen were found to be consistent with PA.

Representative examples of each of these two atypical patterns are highlighted in the representative cases that follow.

### Case 1

**Figure 1:**
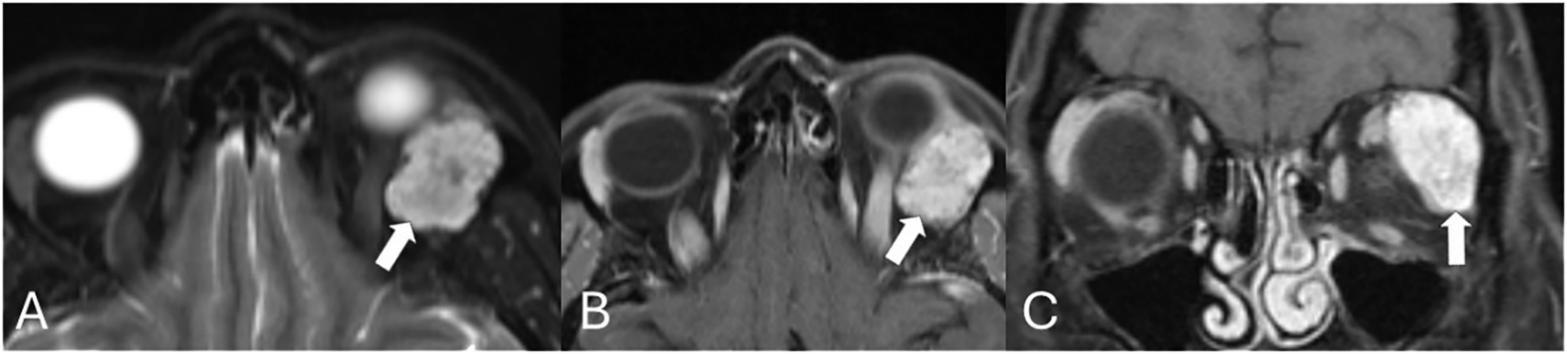
A 45-year-old woman with a left lacrimal pleomorphic adenoma. **(A)** Axial T2 MRI shows a multilobulated iso- to hyperintense appearance of the lacrimal mass (arrow). **(B, C)** Axial and coronal T1 post-contrast MRI shows an oval-shaped mass with heterogeneous enhancement that extends posteriorly in the orbit without bony involvement (arrows).

A 45-year-old woman presented with left eye proptosis and hypoglobus. There was also a mild abduction deficit on the left side. She endorsed intermittent pain in the left eye and denied any visual changes or double vision. On exam, her visual acuity was 20/25 in both eyes. There were no other significant findings on examination. An MRI of orbit showed a multilobulated mass in the lacrimal gland fossa that was difficult to differentiate between a PA and carcinoma (**Figure 1**). She had a left anterior orbitotomy and gross total resection of the left lacrimal gland mass. The pathology report confirmed the diagnosis of cellular PA (a PA predominantly composed of myoepithelial cells) of approximately 3.3 cm in size.

### Case 2

**Figure 2:**
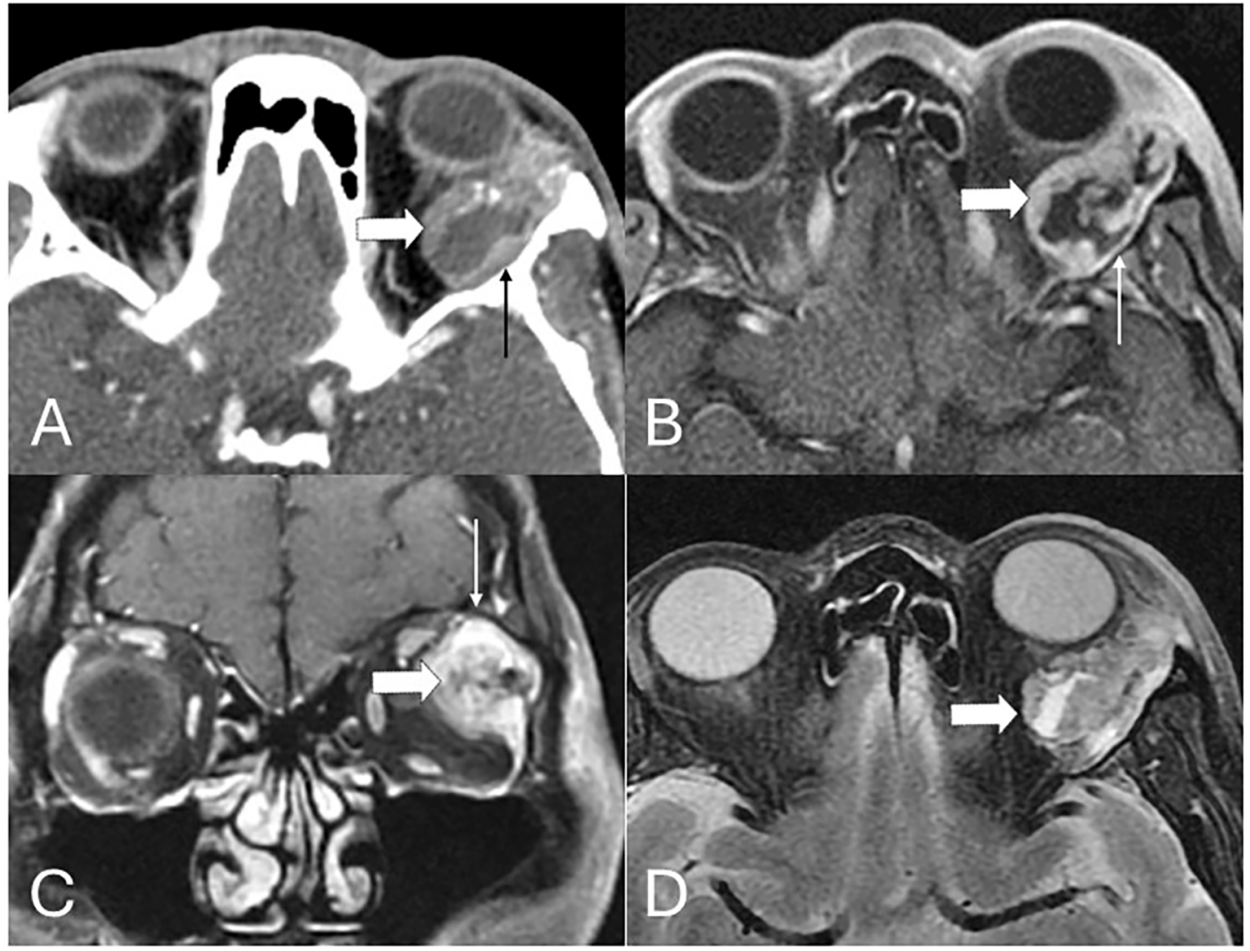
A 57-year-old man with a left lacrimal pleomorphic adenoma. **(A)** Axial CT with contrast shows a cystic and solid lacrimal mass with calcification (white arrow). Note the remodeling of the lateral orbital wall (small black arrow). **(B, C)** Axial and coronal T1 post-contrast MRI shows an oval-shaped mass with heterogeneous enhancement (large white arrows) and remodeling of the orbital wall and roof, respectively (small white arrows). **(D)** Axial T2 MRI shows a cystic and solid appearance of the lacrimal mass (arrow).

A 57-year-old man presented to an outside ophthalmologist with left-sided proptosis. A CT scan showed a left lacrimal gland mass that was non-homogeneous, measuring 2.9 × 2.1 cm without significant erosion of the lateral orbital wall; there was slight remodeling (**Figure 2A**). The patient underwent an incisional biopsy and partial removal of the lesion at an outside facility. The pathology review was consistent with PA. The patient was then referred to our center. An MRI of the orbit demonstrated a heterogenous lesion in the lateral left orbit measuring approximately 3.3 × 1.8 × 2.1 cm (**Figures 2B–D**).

The patient complained of some orbital pain. On exam, there was left proptosis and fullness of the left upper lid. There were also some choroidal striae due to mass effect from the lesion. Visual acuity and color vision were normal in both eyes. The rest of the examination was within normal limits. The patient had a gross total resection of the lacrimal gland mass. The pathology report showed a cellular PA, approximately 3.0 cm in diameter.

### Case 3

**Figure 3:**
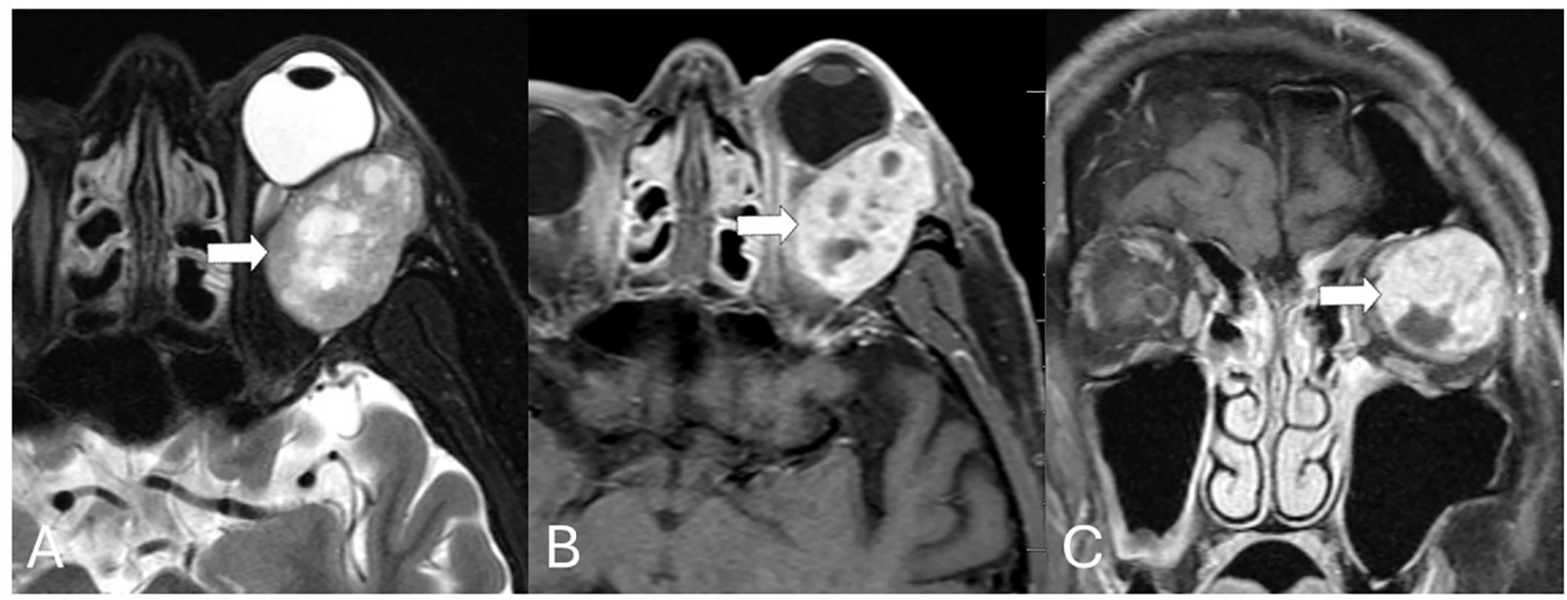
A 55-year-old man with a left lacrimal adenoid cystic carcinoma. **(A)** Axial T2 MRI shows a cystic and solid appearance of the left lacrimal mass (arrow). **(B, C)** Axial and coronal T1 post-contrast MRI shows an oval-shaped mass with enhancement of the solid component (arrows).

A 55-year-old man presented for further evaluation and management of progressive left proptosis and diplopia for the past 3 years. He also complained of “pressure sensation” for the last 3 months but denied orbital pain. On examination, the visual acuity was 20/30 in the right eye and 20/80 in the left eye. There was severe proptosis as well as an abduction deficit in the left eye. A dilated retinal exam showed choroidal striae. MRI revealed a well-circumscribed, partially cystic left lacrimal gland mass (**Figure 3**). He had gross total resection of the left lacrimal gland mass. The pathologic evaluation of the surgical specimen confirmed a diagnosis of adenoid cystic carcinoma, predominantly of cribriform pattern. The patient subsequently completed adjuvant radiation therapy with concurrent chemotherapy.

### Case 4

**Figure 4:**
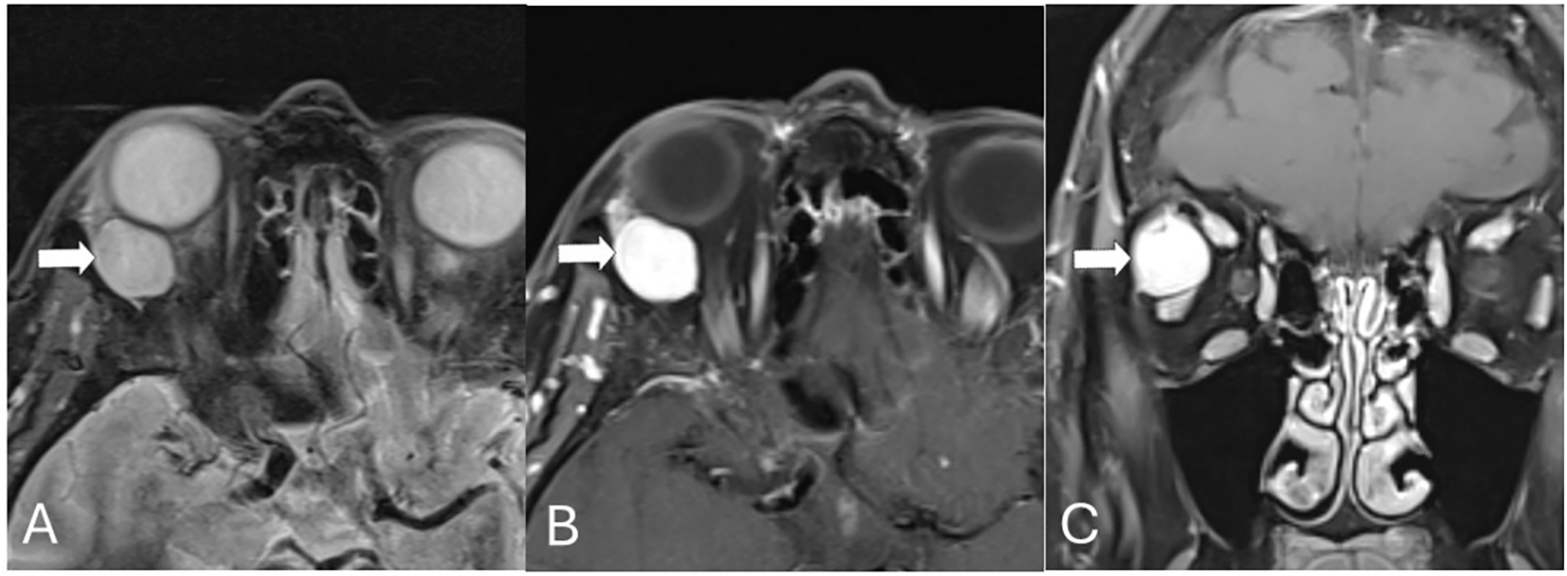
A 70-year-old woman with a right lacrimal adenoid cystic carcinoma. **(A)** Axial T2 MRI shows a homogeneously hyperintense appearance of the right lacrimal mass (arrow). **(B, C)** Axial and coronal T1 post-contrast MRI shows an oval-shaped mass with homogeneous enhancement (arrows).

A 70-year-old woman had an MRI of the orbit for further evaluation of headaches. A right lacrimal gland mass was found as an incidental finding on MRI (**Figure 4**). On exam, she had right eye proptosis without signs of compressive optic neuropathy. She had full motility and no double vision. The visual acuity was normal in both eyes. The patient complained of some “pressure” in the right eye but no pain.

She had gross total resection of the right lacrimal gland mass for the presumed preoperative diagnosis of PA. The pathology report was consistent with a diagnosis of adenoid cystic carcinoma, predominantly of cribriform subtype. The patient subsequently completed adjuvant radiation therapy with concurrent chemotherapy.

## Discussion

Our findings suggest some overlap in radiologic features between PAs and lacrimal gland carcinomas. This finding is of great practical importance since the decision regarding the type of initial surgical intervention, incisional biopsy vs. en-bloc gross total resection of the lacrimal gland mass, largely depends on the preoperative imaging characteristics of the lacrimal gland lesion. Our study of 101 cases of epithelial lacrimal gland lesions found that, approximately 20% of the time, a well-circumscribed round lacrimal gland mass ends up being a lacrimal gland carcinoma upon pathologic evaluation of the surgical specimen, and more than 25% of the time, a multilobular large heterogenous lacrimal gland mass that is suspected to be a lacrimal gland carcinoma ends up being a PA upon pathologic evaluation of the surgical specimen. These findings, especially in the era of selective offering of eye-sparing surgery to most patients with lacrimal gland carcinoma, highlight the importance of erring on the side of gross total resection of the entire lacrimal gland mass as an initial surgical procedure, whenever possible. This is particularly the case for large heterogenous multilobular lesions that may represent a PA despite their irregular shape or multilobular nature as demonstrated nicely in Case 2. Previous publications have described the typical imaging findings associated with a malignant lacrimal gland tumor (3, 11), but little has been written about the atypical presentations and how to best mitigate such cases. Given the risk of multifocal benign or malignant recurrence of PAs if excised incompletely, it seems prudent to err on the side of gross total resection for most well-circumscribed lesions of the lacrimal gland that lack aggressive radiologic features. Some of these well-circumscribed lesions will end up being adenoid cystic carcinoma or other types of lacrimal gland carcinomas upon pathologic evaluation of the surgical specimen (as seen in Case 4) and may require additional adjuvant treatments. It is also prudent to carry out gross total resection of a multilobular heterogeneous large lacrimal gland lesion that may not be typical for a PA (such as Case 2 in this report) given that incomplete excision or an incisional biopsy of lacrimal gland PA can lead to devastating outcomes such as multifocal seeding, increased risk of local recurrence, and potential malignant transformation (5, 6).

An incisional biopsy may be the most appropriate first step for lacrimal gland lesions that have the typical imaging findings for lacrimal gland carcinoma, for example, an obvious posterior extension toward the orbital apex and superior orbital fissure, the so-called “wedge sign (3). Definitive surgical treatment for lacrimal gland carcinoma still would have to be carried out by offering more surgery after the diagnosis is established, and in the last decade, for the majority of cases of lacrimal gland carcinoma, if they can be fully resected without sacrifice of critical structures such as extraocular muscles or optic nerve, eye-sparing surgery could be offered. Any residual microscopic disease is subsequently addressed with adjuvant high-dose radiation therapy (10, 12). In the last decade, the literature on this topic supports offering eye-sparing surgery followed by adjuvant radiation therapy for most patients with lacrimal gland carcinoma including adenoid cystic carcinoma (10, 12–15).

Fine-needle aspiration biopsies are commonly done for salivary gland tumors but have not been used in clinical practice for lacrimal gland tumors, and the jury is out whether FNA of a lacrimal gland mass would lead to accurate diagnosis and whether it would be associated with a risk for recurrence in the case of PA of the lacrimal gland (16). Pain as a diagnostic sign of lacrimal gland adenoid cystic carcinoma (LGACC) or any type of lacrimal gland carcinoma is not necessarily a reliable clinical symptom. In a recent study of 53 cases of LGACC, pain was present in only 30% of the cases (12).

Some authors have advocated for incisional or small biopsies of a lacrimal gland mass to keep “the lacrimal artery” with the idea that this would allow for better and more effective delivery of neoadjuvant intra-arterial chemotherapy (IAC) prior to orbital exenteration and post-op adjuvant chemotherapy protocol for LGACC (17, 18). Yet, there are no published data such as angiographic evidence of whether the presence or absence of an intact lacrimal artery was correlated with long-term disease control in the published reports of IAC for LGACC (17, 18).

In conclusion, our report suggests that the historical teachings for typical preoperative imaging findings for lacrimal gland PA vs. lacrimal gland carcinoma do not hold in every case and approximately one-quarter of cases will demonstrate atypical imaging features. The main concern is incomplete excision of a PA that may have imaging findings suggestive of carcinoma. In these overlapping atypical cases, it seems prudent to err on the side of caution and do a complete excision of the lacrimal gland mass if the lesion is surgically resectable without an orbital exenteration and without sacrifice of important orbital structures such as extraocular muscles or optic nerve. A limitation of our study is its retrospective nature and the fact that the study period spanned several decades; however, given the rare nature of lacrimal gland epithelial tumors, a long period of accrual is inevitable and prospective randomized studies are not feasible.

## Data Availability

The datasets will not be made available publicly. Requests to access the datasets should be directed to besmaeli@houstonmethodist.org.
